# Editorial: Pluripotent Cells for Stroke: From Mechanism to Therapeutic Strategies

**DOI:** 10.3389/fncel.2021.738240

**Published:** 2021-08-04

**Authors:** Yujie Chen, Yao Yao, John H. Zhang, Shilei Hao

**Affiliations:** ^1^Department of Neurosurgery and State Key Laboratory of Trauma, Burn and Combined Injury, Southwest Hospital, Third Military Medical University (Army Medical University), Chongqing, China; ^2^Chongqing Key Laboratory of Precision Neuromedicine and Neuroregenaration, Third Military Medical University (Army Medical University), Chongqing, China; ^3^Department of Pharmaceutical and Biomedical Sciences, University of Georgia, Athens, GA, United States; ^4^Neuroscience Research Center, Loma Linda University, Loma Linda, CA, United States; ^5^Key Laboratory of Biorheological Science and Technology, Ministry of Education, College of Bioengineering, Chongqing University, Chongqing, China

**Keywords:** stroke, pluripotent cell, stem cell, pericyte, microenvironment, neural circuit

Stroke, an acute central nervous system injury caused by cerebral ischemia or cerebral hemorrhage, is one of the major causes of death and the leading cause of long-term disability worldwide (Dalys and Collaborators, 2017; Wang et al., 2020). Thanks to early diagnosis, mini-invasive surgery, and advanced intensive care support, stroke survival rate has increased dramatically (Kim et al., 2020; Wang et al., 2020). Nevertheless, stroke survivors usually suffer from sequelae of neurological impairments and psychiatric disorders, which affect their daily functionality and working capacity. Although extensively studied, the molecular mechanisms underlying stroke pathophysiology remain not fully understood and innovative rehabilitation therapies for neural circuit remodeling after stroke are urgently needed.

Recently, pluripotent cell-based approaches have attracted more attention from scientists and physicians due to their possible neuroprotective and restorative effects on stroke. One major challenge, however, is that injury-induced microenvironments usually lead to obstruction in directional differentiation of pluripotent cells, and failure in reconstruction of neural circuits. Additionally, the conventional neurobehavioral evaluation and diffusion-weighted magnetic resonance imaging (MRI-DWI) cannot monitor the evolution of pluripotent cells differentiation. As a consequence, various pluripotent cell-based strategies exhibited uncertain neuroprotective efficacy in previous clinical trials.

This Research Topic contains 12 manuscripts, highlighting current understanding and future directions in pluripotent cell-based therapies in stroke. Due to their proliferation and differentiation potential, both endogenous and exogenous stem cells are candidates for neural regeneration and neural circuit remodeling. Liu et al. summarize the mechanisms, processes, and challenges of using stem cells in stroke treatment. They conclude that specific cell types, dosages and routes, and other issues for stem cell-based approaches still need to be optimized in the near future. Zhang et al. systemically review stem cell-based therapies for experimental ischemic stroke. They conclude that stem cell-based therapies are able to improve neurological function and reduce infarct volume, but further clinical studies are needed to verify these neuroprotective effects. Mesenchymal stem cells (MSCs) have been proven effective in ischemic stroke (Li et al.), intracerebral hemorrhage (Gong et al.), and neurodegeneration diseases, such as Alzheimer's Disease (Yan et al.). Islam et al. also report that human neuroepithelial precursor cells (cNEPs) grafted early after stroke are able to improve functional recovery. Traditional Chinese medicine monomers, active components extracted from Chinese herbs, also exhibit the potential to activate proliferation and neurogenesis of neural stem cells after stroke (Wang et al.). Mechanistic studies show that canonical Wnt/β-catenin signaling and other molecules play a pivotal role in neural stem/progenitor cell differentiation (Kriska et al.).

Due to the limitations of neural stem cells, induced pluripotent stem cells (iPSCs) have been used as an alternative cell type in stroke treatment. iPSCs, produced by introducing specific transcription factors into somatic cells, exhibit similar differentiation potential as embryonic stem cells. Duan et al. summarize the current applications of iPSC therapy in ischemic stroke. In addition, inspired by iPSC and *in vivo* reprogramming technology, endogenous and reactive glial cells have been *in situ* converted to functional neurons for the treatments of stroke, neurodegenerative diseases, retinal diseases, and trauma in central nervous system (Wang et al., 2021).

In the concept of neuro-vascular network (Zhang et al., 2012), non-neuronal cells are equally important and play an essential role in neural circuit remodeling and neurofunctional recovery. Microglial cells, major immune cells in brain, are influenced by low dose of valproic acid after stroke, possibly via interleukin-6- and galectin-3-mediated extracellular matrix remodeling (Kuo et al.). Gan et al. elucidate the regulatory functions of lncRNAs on angiogenesis after stroke, which are important for vascular reconstruction and blood flow support of neural circuit remodeling. In addition, Cao et al. summarize the functions of pericytes as perivascular multi-potent cells and an important component of the blood-brain barrier in the central nervous system (Gautam and Yao, 2018). They discuss how pericyte dysfunction affects the pathophysiological processes of stroke. A subset of pericytes have been shown to trans-differentiate into neurons in the damaged tissue (Karow et al., 2018).

In summary, there are 12 outstanding manuscripts, including both original studies and comprehensive reviews, in this Research Topic. We summarize recent findings and challenges of pluripotent cell-based therapies in stroke, and propose to explore the mechanisms of directed differentiation and optimize pluripotent cells products & *in vivo* tracking techniques in further studies.

## Author Contributions

All authors listed have made a substantial, direct and intellectual contribution to the work, and approved it for publication.

## Conflict of Interest

The authors declare that the research was conducted in the absence of any commercial or financial relationships that could be construed as a potential conflict of interest.

## Publisher's Note

All claims expressed in this article are solely those of the authors and do not necessarily represent those of their affiliated organizations, or those of the publisher, the editors and the reviewers. Any product that may be evaluated in this article, or claim that may be made by its manufacturer, is not guaranteed or endorsed by the publisher.

## References

[B1] Dalys G.B. D. Collaborators H. (2017). Global, regional, and national disability-adjusted life-years (DALYs) for 333 diseases and injuries and healthy life expectancy (HALE) for 195 countries and territories, 1990-2016: a systematic analysis for the Global Burden of Disease Study 2016. Lancet 390, 1260–1344. 10.1016/S0140-6736(17)32130-X 28919118 PMC5605707

[B2] Gautam J. Yao Y. (2018). Roles of pericytes in stroke pathogenesis. Cell Transplant. 27, 1798–1808. 10.1177/0963689718768455 29845887 PMC6300777

[B3] Karow M. Camp J. G. Falk S. Gerber T. Pataskar A. Gac-Santel M.. (2018). Direct pericyte-to-neuron reprogramming via unfolding of a neural stem cell-like program. Nat. Neurosci. 21, 932–940. 10.1038/s41593-018-0168-3 29915193 PMC6319609

[B4] Kim J. Thayabaranathan T. Donnan G. A. Howard G. Howard V. J. Rothwell P. M.. (2020). Global stroke statistics 2019. Int. J. Stroke 15, 819–838. 10.1177/1747493020909545 32146867

[B5] Wang Q. Li W. Lei W. Chen W. Zheng J. Xiang Z.. (2021). *In situ* neuroregeneration: frontiers and therapeutic prospects (in Chinese). Sci. Sin. Vitae. 51. 10.1360/SSV-2021-0024

[B6] Wang Y. J. Li Z. X. Gu H. Q. Zhai Y. Jiang Y. Zhao X. Q.. (2020). China Stroke Statistics 2019: a Report From the National Center for Healthcare Quality Management in Neurological Diseases, China National Clinical Research Center for Neurological Diseases, the Chinese Stroke Association, National Center for Chronic and Non-communicable Disease Control and Prevention, Chinese Center for Disease Control and Prevention and Institute for Global Neuroscience and Stroke Collaborations. Stroke Vasc. Neurol. 5, 211–239. 10.1136/svn-2020-000457 32826385 PMC7548521

[B7] Zhang J. H. Badaut J. Tang J. Obenaus A. Hartman R. Pearce W. J. (2012). The vascular neural network–a new paradigm in stroke pathophysiology. Nat. Rev. Neurol. 8, 711–716. 10.1038/nrneurol.2012.210 23070610 PMC3595043

